# An active machine learning approach for optimal design of magnesium alloys using Bayesian optimisation

**DOI:** 10.1038/s41598-024-59100-9

**Published:** 2024-04-09

**Authors:** M. Ghorbani, M. Boley, P. N. H. Nakashima, N. Birbilis

**Affiliations:** 1 Department of Materials Science and Engineering, Monash University, Melbourne, VIC 3800 Australia; 2 Faculty of Engineering, Science and the Built Environment, Deakin University, Waurn Ponds, VIC 3125 Australia; 3 Faculty of Information Technology, Monash University, Melbourne, VIC 3800 Australia

**Keywords:** Mg alloys, Alloy design, Machine learning, Bayesian optimisation, Sequential design strategy, Materials science, Structural materials, Theory and computation

## Abstract

In the pursuit of magnesium (Mg) alloys with targeted mechanical properties, a multi-objective Bayesian optimisation workflow is presented to enable optimal Mg-alloy design. A probabilistic Gaussian process regressor model was trained through an active learning loop, while balancing the exploration and exploitation trade-off via an acquisition function of the upper confidence bound. New candidate alloys suggested by the optimiser within each iteration were appended to the training data, and the performance of this sequential strategy was validated via a regret analysis. Using the proposed approach, the dependency of the prediction error on the training data was overcome by considering both the predictions and their associated uncertainties. The method developed here, has been packaged into a web tool with a graphical user-interactive interface (GUI) that allows the proposed optimal Mg-alloy design strategy to be deployed.

## Introduction

Magnesium (Mg) alloys continue to garner attention due to their potential for enhancing energy efficiency in numerous applications^1^. The strength-to-weight ratio of Mg alloys makes them appealing for use in weight-sensitive applications such as the aerospace, automotive and electronic (3C) industries^2–4^. Despite such potential, the more extensive application of Mg alloys remains—in part—constrained by their balance of mechanical properties, including the attainment of strength with appropriate ductility^1^. Whilst researchers continue to address such issues by alloying and manufacturing processes^1,2^; the potentially (very) wide range of possible alloy compositions and processing parameters presents an empirical challenge in achieving the optimal design for specific applications.

One promising approach for expediting the discovery of metallic alloys with target mechanical properties is by using machine learning^5–8^. Machine learning (ML) accelerates new materials discovery by reducing the time and cost required for traditional trial-and-error approaches^6,9,10^, and utilising large datasets, advanced algorithms, and computational methods. This enables the acceleration of optimal alloy identification. In particular, so-called active machine learning approaches, which combine human expertise with iterative model refinement, have demonstrated great potential in reducing the experimental burden and maximising the search efficiency in materials design^11–14^. Bayesian optimisation and adaptive design are methods following an active ML strategy, which require goal-directed iterative feedback^6,15,16^.

Bayesian optimisation is an ML-based method for optimising computationally ‘expensive’ black-box functions; where the objective function is not explicitly known and must be evaluated through time-consuming processes such as experiments or simulations^17^. To date, Bayesian optimisation has been successful in a wide range of applications, including drug discovery, robotics, and materials science^18–25^. In the context of Mg alloys, for the first time, Bayesian optimisation is considered to identify the composition and processing conditions that lead to desired mechanical properties, such as strength and ductility. Bayesian optimisation can optimise multiple properties simultaneously, which is particularly useful in the design of metallic alloys, where different applications can have conflicting requirements. Specifically, in the case of Mg-alloys where increasing strength can often lead to a reduction in ductility, Bayesian optimisation can help identify the optimal trade-off between conflicting properties. The use of ML-based Bayesian optimisation in the design of metallic alloys remains a relatively new field with many challenges and opportunities for future research, despite Bayesian optimisation having been utilised quite heavily in other domains over the past decade. One challenge is the need for high-quality data, especially when experiments and simulations are expensive or time-consuming. Another challenge is the need for accurate models that can, in the context of alloys, capture complex relationships between composition, structure, processing conditions, and resultant mechanical properties.

The work herein proposes an active machine-learning approach for the optimal design of Mg alloys, utilising Bayesian optimisation. Whilst the approach employed in the present work is elaborated upon in the accompanying methods section, Bayesian optimisation is particularly relevant because the method employs probabilistic models to guide the search for a ‘best solution’ in a noisy parameter space of high dimensionality. By iteratively selecting the most informative experiments, Bayesian optimisation facilitates the exploration and exploitation of the Mg alloys design space, sampling more efficiently, and potentially leading to the discovery of promising alloy compositions with enhanced mechanical properties^26^. The objective of the present study is to develop an active learning framework that combines Bayesian optimisation with a comprehensive dataset of Mg alloys. The details of the data collection for Mg alloys have been provided in the previous work of the authors^9^. The framework leverages available data, expert knowledge, and accurate random forest (RF) models that have been trained with that Mg alloys data^10^. The proposed approach can enhance the efficiency of materials design by iteratively improving the model's predictive capabilities while simultaneously optimising the alloy's mechanical properties. Relying on the performance of those accurate and robust RF models within the proposed active learning loop can reduce the need for the expensive and time-consuming experiments.

Furthermore, the present study provides insights into the underlying relationships between alloy composition, processing parameters, and properties. By illuminating these complex interactions, the proposed active ML approach can accelerate the design of Mg alloys. Some fundamental concepts of ML and the principles of Bayesian optimisation are first introduced. Thereafter, the specific application of ML and Bayesian optimisation in the design of Mg alloys, including the prediction of mechanical properties, is elaborated upon. Critically, what is believed to be the first ‘user tool’ for digitally optimised Mg-alloy design is presented. The challenges and potential opportunities for future research in this field are also discussed.

## Methods

### Dataset

The alloy dataset utilised in this study includes three key categories, namely: thermomechanical processing conditions; chemical composition; and mechanical properties. The production routes and processing treatments for the alloys are comprised of a range of casting or thermomechanical processes (including heat treatments). To classify the alloys in a rational manner, these different production routes were encoded into one of six mutually exclusive categories by human experts. Furthermore, using the one-hot encoding method, the categorical processing data was encoded onto vectors of zeros and ones. The categories of processing designation that capture the alloys in the database are summarised in Table 1.

**Table 1:** Summary of the six categories of production route and processing treatments for the Mg-alloys in the compiled database^9^.

Shortform designation	Processing type	Description
Sand cast	Refers to alloys that have been cast into their near-net shape and cast by a process that has a slow cooling rate	This category is notionally sand-cast alloys, or alloys cast and cooled in place
HPDC	Refers to alloys that have been cast into their near net-shape but using a process that has rapid cooling	This category is notionally high pressure die-cast (HPDC) alloys, or those alloys cast into a cooled mould
Cast + HT	This designation was given to any alloy that has been cast (with either slow or rapid cooling) and then subsequently “heat treated” to obtain specific properties	This category includes both sand cast and HPDC prepared alloys that have been heat treated (e.g., to attain specific tempers)
Extruded	Refers to any alloy (regardless of casting process) that has been processed to include an extrusion step	These alloys may or may not be heat treated after extrusion
ECAE/ECAP	Refers to any alloy (regardless of casting process) that has been processed to include equal channel angular extrusion or pressing (ECAE/ECAP) in its processing	These alloys were notionally produced at a small scale
Wrought	This designation was given to any alloy that has undergone wrought processing (except for the alloys that have been proceed by extrusion or ECAE/ECAP)	These alloys have undergone some wrought operation (e.g., rolling) and may or may not have been heat treated

It is conceded that the shortform designations in Table 1 have truncated the many subvariants of processing which may have been carried out. This is a deliberate trade-off between having a rational number of processing conditions, and each processing condition being unique enough to disambiguate from alloys falling into two processing categories. The 916 unique Mg alloys in the original dataset incorporate at least one or more of 30 alloying elements. The elements in the alloy compositions including Mg, along with their range (wt%), mean, and standard deviation are summarised in Table 2.

**Table 2:** Elements in the Mg alloy database utilised for this study, including their range (minimum and maximum (wt. %), mean, and standard deviation. The elements have been sorted based on their prevalence in the database^9^.

Element	Range (wt%)	Mean	Standard deviation
Mg	65.0—100.0	91.96	4.78
Zn	0.002—14.3	2.4	2.61
Al	0.004—20.0	5.69	3.41
Mn	0.004—2.0	0.42	0.36
Zr	0.01—3.0	0.56	0.3
Nd	0.01—8.1	1.57	1.34
Ce	0.01—3.9	0.88	0.74
La	0.01—6.0	1.22	1.24
Y	0.2—19.0	3.63	2.31
Cu	0.001—4.0	0.23	0.67
Si	0.005—13.0	1.37	3.23
Gd	0.19—25.6	9.38	4.5
Ca	0.04—15.0	1,59	2.41
Pr	0.005—1.8	0.18	0.49
Ni	0.004—11.0	0.74	2.54
Fe	0.001—0.0128	0.01	0.0
Li	0.2—14.0	6.82	4.27
Be	0.0001—0.0008	0.00	0.0
Sn	0.25—9.6	3.66	2.69
Th	0.7—3.3	2.13	0.94
Ag	1.0 – 6.0	2.33	1.1
Sb	0.15—1.0	0.49	0.3
Er	0.5—6.0	3.08	2.34
Dy	0.27—12.0	3.86	5.08
Yb	2.0 – 3.0	2.12	0.35
Bi	0.5—3.0	1.33	0.98
Sr	0.3—2.5	1.22	1.03
Ga	2.0 – 5.0	2.8	1.3
Sc	0.01—15.2	5.35	7.1
Tb	0.5—19.7	10.1	13.58
Ho	1.0—1.4	1.2	0.28

The mechanical properties of alloys in the dataset have been restricted to the yield strength (YS), ultimate tensile strength (UTS), and elongation / ductility along with their lower and upper bounds, mean, and standard deviation are provided in Table 3.

**Table 3:** Lower and upper bounds, mean, and standard deviation of the mechanical properties for the Mg alloys in the dataset^9^.

Target property	Range	Mean	Standard deviation
Yield strength, YS (MPa)	21.0 – 610.0	206.17	106.93
Ultimate tensile strength, UTS (MPa)	52.0 – 710.0	275.88	106.57
Ductility (%)	0.23 – 65.2	9.25	8.79

The compiled and formatted database is publicly accessible through the following link: https://github.com/katrina-coder/Magnesium-alloys-database^9^.

### Bayesian optimisation

Bayesian optimisation is an ML-based technique that may be applied to solve an optimisation problem in which the objective function is continuous, expensive to evaluate, derivative-free and ‘black-box’^27–29^. In alloy design and discovery, iterative trial and error experiments make evaluating the alloy properties costly and time-consuming. The composition-process-property relationship of an alloy (or alloys) as a continuous objective function is unknown and often non-convex, non-linear, and high dimensional (and may be considered a black-box problem). In addition, the target property (*f*(x)) is being observed without its derivatives^30,31^. As a result, to discover new Mg alloys, a Bayesian optimiser was developed that consists of two main components. The first component was a surrogate function (probabilistic model) that estimates the mechanical property. In this case, a gaussian process regressor model (GPR) computed a posterior probability distribution based on Bayes’ rule^32,33^. The distribution included the function estimate and associated uncertainty around the estimation^34–36^. The second component of the optimiser was an acquisition function to specify the next candidate sample and evaluate its target property based on what was known (so far) from the posterior distribution. An acquisition function made this decision by balancing the exploration of the uncertain regions and exploitation of the regions with known higher target values^24,37^.

**Figure 1:**
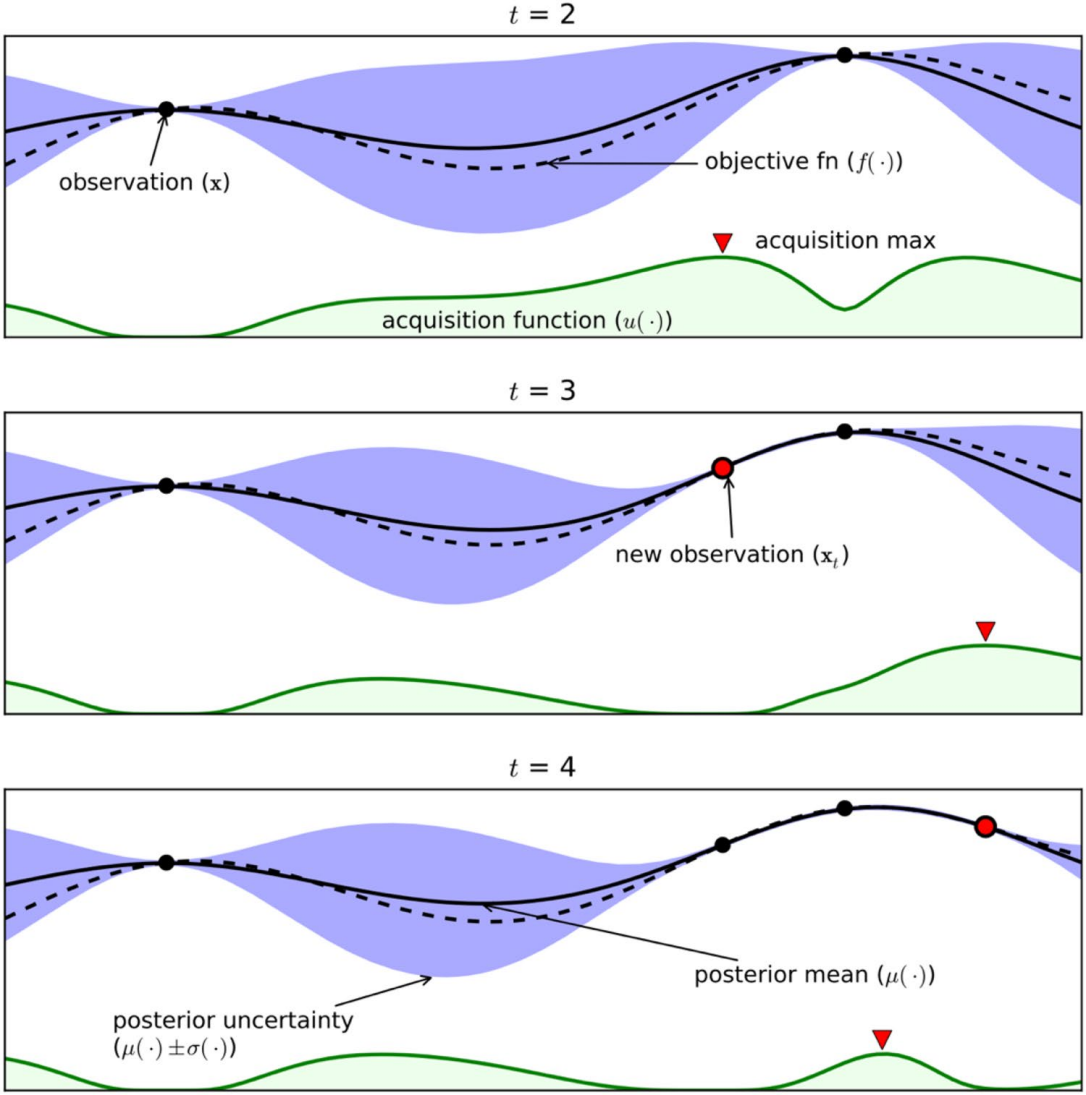
Schematic workflow of active learning over three iterations (iterations number 2–4). The dashed black lines show the objective function, and the solid black lines show the posterior mean of the Gaussian process (GP) approximation. The purple regions indicate the posterior uncertainty of the GP surrogate model’s prediction. The green areas represent the acquisition (utility) functions that balance the exploitation and exploration within the search space. Note that the acquisition function value is high where the prediction of the objective function by GP is high (exploitation) and where the prediction uncertainty is high (exploration) — areas with both attributes are sampled first^24^.

The probabilistic model was updated as new datapoints were acquired, and the acquisition function used the current state of the model to determine the next point to evaluate. The goal was to find an optimum of the function with the minimum number of evaluations. The suggested new alloy was added to the training data and the processes of modelling and querying the next sample were repeated. The optimiser learned dynamically (active learning) in that sense, and the optimal composition was expected to be found within a certain number of iterations (Fig. 1).

### Regret analysis

Regret analysis is used to quantify the performance of an optimisation algorithm. It measures the difference between the value of the objective function at the ideal optimal point and the value of the objective function at the point obtained by the algorithm. The regret is usually expressed as a function of the number of evaluations or the computation time. Regret analysis is useful for comparing different optimisation algorithms and for determining the asymptotic behaviour of the algorithms. It provides a measure of the trade-off between exploration and exploitation in the optimisation process. In Bayesian optimisation, regret analysis is used to quantify the performance of the algorithm, and to determine the optimal number of function evaluations required to achieve a certain level of performance.

To validate the performance of the developed sequential optimiser for our Mg-alloy dataset, a regret analysis was implemented as defined in Eq. 1.

1 $$r\left(x\right)= f{(x}^{*})-f\left(x\right),$$

where $f(x)$ was known from the value of the target property at the datapoint $x$ which was suggested by the optimiser as the next candidate. While $f{(x}^{*})$ was the optimal value of the target property at the datapoint ${x}^{*}$ that was expected to be found by the optimiser within the entire alloy data ($x\in X$) as defined in Eq. 2.

2 $${x}^{*}=\underset{\mathit{x }\in \mathit{ X}}{\mathit{argmax}}\left(f\left(x\right)\right),$$

First, the gaussian model was trained with a random set of 20 initial datapoints and the next candidates were extracted from the remaining data through 100 iterations. Then, using the known maximum value of the target property within the entire dataset from Eq. 2, the regret value was calculated for each iteration. In can be summarised as defining the ideal value of the target properties ${f\left(x\right)}^{*}$ and regret value within each iteration as follows in Eqs. 3 and 4.

3 $${f\left(x\right)}^{*}=\underset{x \in X}{max}(f(x)),$$

4 $$r\left(x\right)= {f\left(x\right)}^{*}-f\left(x\right),$$

This regret value showed the difference between the real maximum target property value and the maximum target property value determined by the optimiser from the generated samples^38^.

### Multi-objective Bayesian optimisation

#### Method evaluation based on “simulated results”

The validated Bayesian optimiser was used to simultaneously maximise the multiple mechanical properties of Mg alloys. To do this, a multi-objective optimisation problem was defined as follows:

5 $${f\left({\text{x}}\right)}^{*}=\underset{x \in \mathrm{ X}}{{\text{max}}} {\prod }_{{\text{i}}=1}^{{\text{n}}}{f}_{i}\left(x\right),$$

where ${f}_{i}\left(x\right)$ represents the i-th objective function evaluated at input variables via successive queries of $x\in X$^39–41^.

First, a gaussian probabilistic model (GP) was trained as the surrogate function with the target properties of strength and ductility from Mg-alloy data. This can be expressed as follows:

6 $$f\left({\text{x}}\right)\sim {\text{GP}}(\mu \left(x\right), k\left(x,{x}{\prime}\right)),$$

where *µ*(x) is the mean and *k*(*x*,*x*') is the covariance that were computed based on the kernel function definition^42–45^.

Then, new alloys were "discovered" using an acquisition function based on the posterior distribution within iterations. In other words, at iteration *t* + 1, using the target property value of the previous sample of ${x}_{t}$, the posterior distribution was computed by Bayesian inference. Thus, to direct the so-called dynamic learning process, specifying the strength and ductility of the "discovered" alloy at each iteration is necessary, followed by updating the training set, re-fitting the model, and querying the next point. To obtain the actual target property values, lengthy and costly alloy-making and mechanical testing are usually required. As an alternative, the mechanical properties estimated by the RF model replaced the actual values. This method is referred to as evaluation based on simulated results.

Two different acquisition functions, namely upper confidence bound (UCB) and mutual information (MI), were evaluated to suggest new alloys. In UCB, the acquisition function (utility) is defined as:

7 $${{\text{UCB}}}_{t}\left(x\right)={\mu }_{t}(x)+k{\sigma }_{t}(x),$$

where *μ*(*x*) is the exploitation term and *σ*(*x*) is the exploration term. *K* is the constant hyperparameter that controls the trade-off between exploration and exploitation^46,47^. The exploitation term estimates the expected reward at a given point based on the current model's predictions, and the exploration term quantifies the uncertainty or lack of knowledge about the true reward at that point. The exploration term encourages the algorithm to explore regions of the search space with high uncertainty, which may contain better solutions, while the exploitation term guides it towards regions with high predicted rewards based on existing knowledge.

While the UCB is a common choice for managing this trade-off, an alternative approach using MI has emerged as a promising technique. Mutual Information is a measure of the dependence between two random variables and has gained attention in the context of Bayesian optimisation as an acquisition function for managing the exploration–exploitation trade-off. MI-based acquisition functions estimate the information gained by acquiring new data at a particular point in the search space. By maximizing the MI, the algorithm aims to explore regions that provide the most valuable information for reducing the uncertainty about the location of the global optimum. In the defined MI, the term that controls the exploration was updated over the iterations by information gained about *f*(*x*) by the query point *x*_*t*_ as follows^48,49^:

8 $${{\text{MI}}}_{t}\left(x\right)={\mu }_{t}(x)+ {\emptyset }_{t}(x),$$

in which, similar to Eq. 7, *μ*(x) was responsible for the exploitation ability of the function but the exploration term was controlled by Ø(x).

9 $${\emptyset }_{t}\left(x\right)= \sqrt{\alpha }(\sqrt{{\sigma }_{t}^{2}\left(x\right)+{\widehat{\gamma }}_{t-1}}-\sqrt{{\widehat{\gamma }}_{t-1}}),$$

which is an increasing function of *σ*^2^(*x*) and empirically controlled by the amount of exploration that has already been done. The more the algorithm has gathered information on *f*, the more it focuses on the optimum^48,50^. The variable *α* is a constant hyperparameter and $\widehat{\gamma }$ is defined as follows:

10 $${\widehat{\gamma }}_{T}={\sum }_{t=1}^{T}{\sigma }_{t}^{2}\left({x}_{t}\right)$$

In both methods, the kernel function of the gaussian model was tuned and the "rat_quad" kernel was selected as the optimum kernel. The hyperparameters kappa and alpha were also tuned with 0.05 and 0.01, respectively.

#### Batch optimisation

Herein the goal was to find a batch of alloys in a single iteration without updating the model by estimated predictions or actual values of mechanical properties^51^. In sequential learning, predictions at each iteration were used due to limitations in assessing the actual values of UTS and ductility.

**Figure 2:**
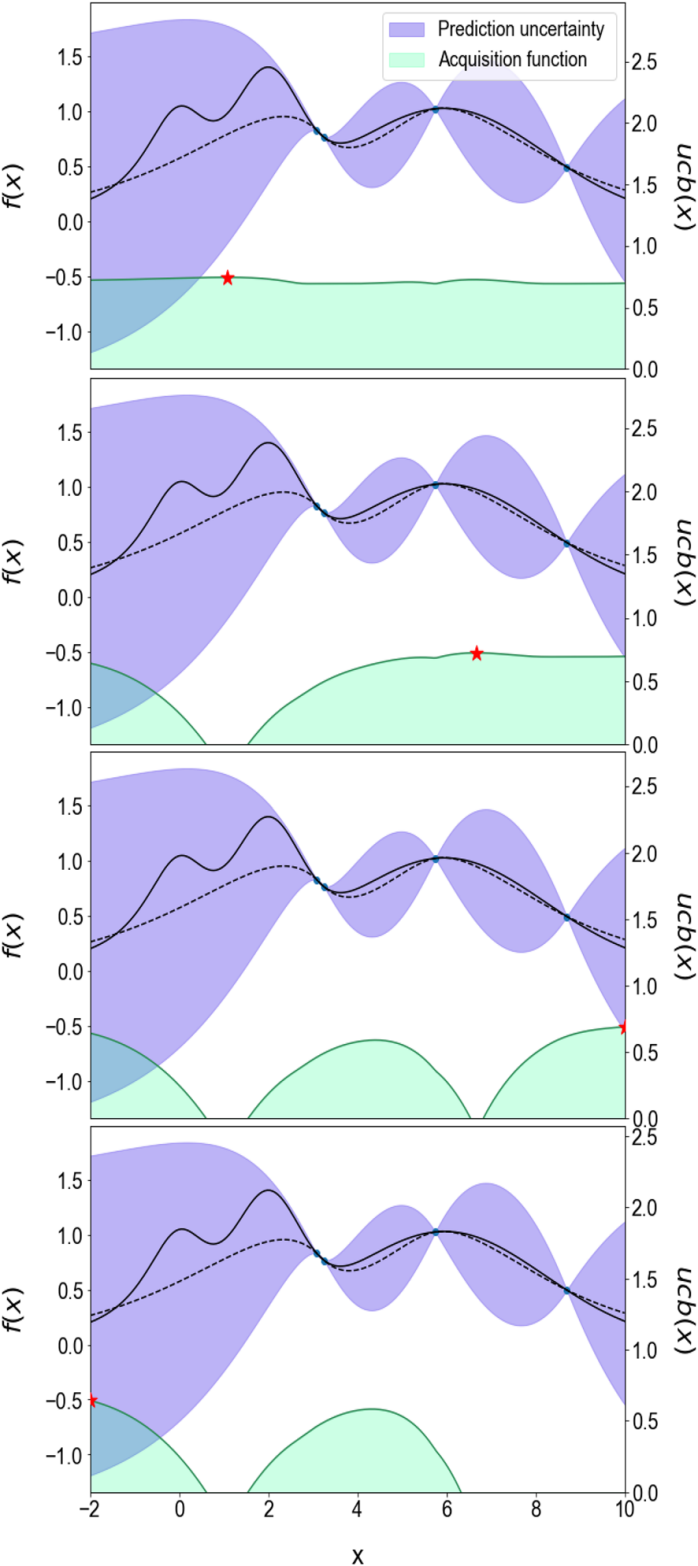
Schematic outline of batch optimisation with a batch size of 4. The figure illustrates the iterative process of selecting optimum points (indicated by red stars) based on the maximisation of the penalised acquisition function, shown as the light green area. The batch optimisation enabled simultaneous evaluation and selection of points in each iteration, achieved by penalising the acquisition function around the previous iteration's red star.

A penalised acquisition function was defined to collect a number of new compositions around the local maximum area of the function by excluding the previous local maximum. A schematic outline of this method for a batch size of 4 is shown in Fig. 2. The purple area shows the probability distribution estimated by the Gaussian regressor model. The first optimum point (red star) is discovered based on maximising the acquisition function (green area) in the first iteration. To obtain the second member of the batch points, the acquisition function is penalised in the second plot at the point around the previous optimum point (previous star, 0.6 < x < 1.5). This process is repeated in the third and fourth plots to discover the next two optimum points.

## Results and discussion

### Regret analysis

**Figure 3:**
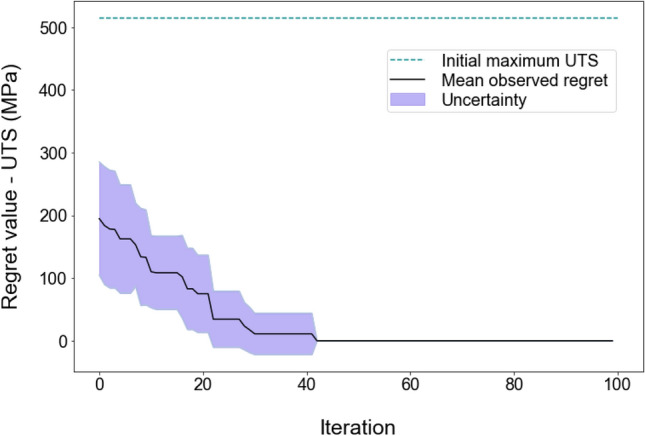
Calculated regret values for UTS optimisation of Mg alloys over 100 iterations by the Bayesian optimiser. The black line and purple area show the average and the standard deviation of regret values respectively, over 10 search trials. The dashed green line represents the mean of the maximum UTS across the 20 random initial datapoints, averaged over 10 repetitions.

**Figure 4:**
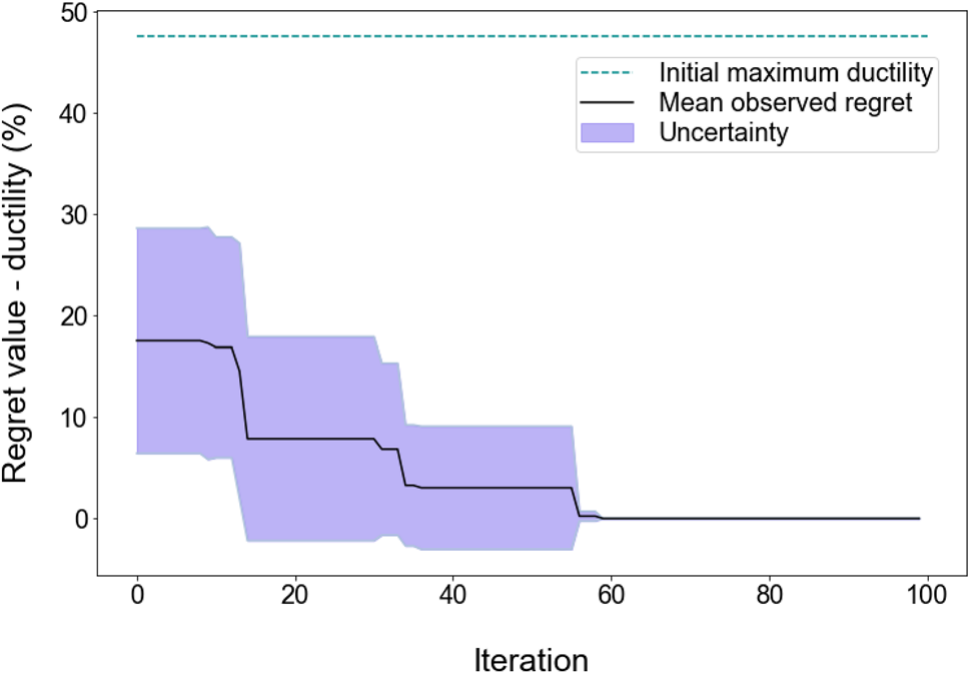
Calculated regret values for ductility optimisation of Mg alloys over 100 iterations by the Bayesian optimiser. The black line and purple area show the average and the standard deviation of regret values respectively, over 10 search trials. The dashed green line represents the mean of the maximum ductility across the 20 random initial datapoints, averaged over 10 repetitions.

Figures 3 and 4 depict the mean (solid black line) and standard deviation (shaded purple area) of regret values over 10 repetitions of searches for the ultimate strength and ductility of Mg alloys, respectively. The results demonstrate that our optimiser successfully identifies the optimal composition for maximised UTS after 42 iterations (Fig. 3) and for maximised ductility after 59 iterations (Fig. 4), at which points that the regret values reach zero. In alignment with the goal of the regret analysis, the optimum is achieved when the regret value is zero, indicating that the optimiser has identified the points with the maximum target properties. The dashed green line represents the mean of the maximum target property across the 20 random initial datapoints, averaged over 10 repetitions. Since the optimiser started to learn from only 20 initial training datapoints, it can be claimed that its performance is efficient enough in real searches where training uses the whole dataset.

The performance of the trained Gaussian regressor models with the whole dataset along with the associated uncertainties are shown in Figs. S1 and S2 for the prediction of UTS and ductility. Both parity plots (actual value in the X-axis and Gaussian probabilistic model predicted value in the Y-axis) show that the model is efficient enough as a surrogate function within the Bayesian optimiser.

### Multi-objective Bayesian optimisation based on “simulated results”

**Figure 5:**
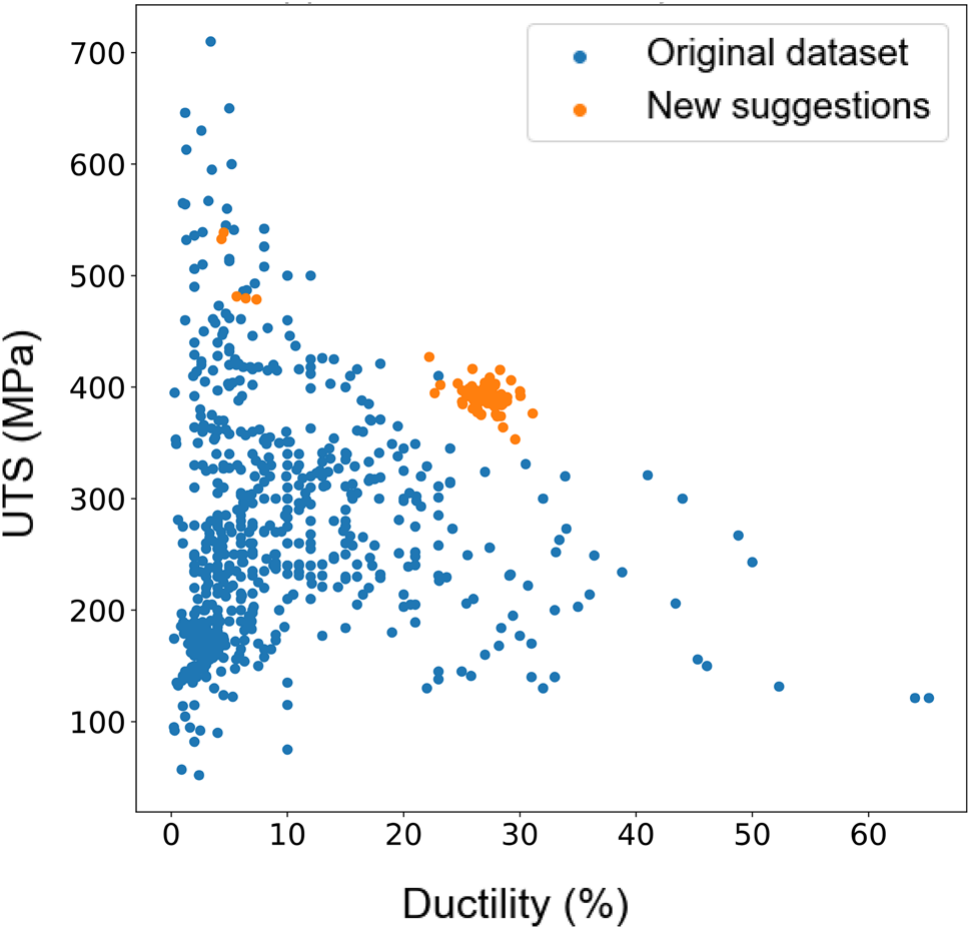
Proposed new Mg alloys (orange dots) and existing alloys (blue dots) are plotted in terms of UTS versus ductility as obtained by the UCB method in Bayesian optimisation.

**Figure 6:**
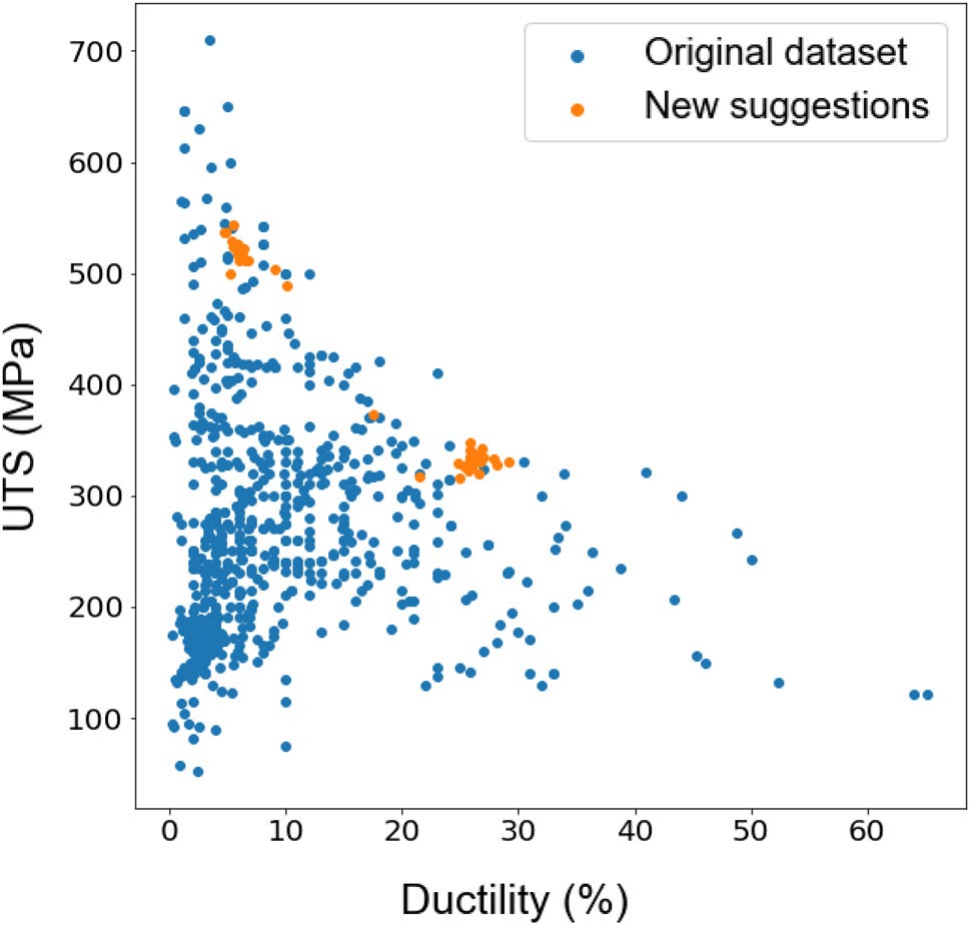
Proposed new Mg alloys (orange dots) and existing alloys (blue dots) are plotted in terms of UTS versus ductility as obtained by the MI method in Bayesian optimisation.

By iteratively selecting the point that maximises the acquisition function, the UCB or MI algorithms gradually balance exploration and exploitation, resulting in an efficient search process. Initially, the algorithms explore different regions of the search space, allowing the probabilistic model to learn and update its predictions based on the observed data. As the algorithms gather more information, the exploitation term becomes dominant, leading to a focus on promising regions and converging towards the global optimum. Predicted UTS and ductility of proposed alloys (orange dots) are shown in Figs. 5 and 6 for the UCB and MI methods respectively. Blue points show the values of mechanical properties for the existing Mg alloys.

#### Exploration–exploitation trade-off

**Figure 7:**
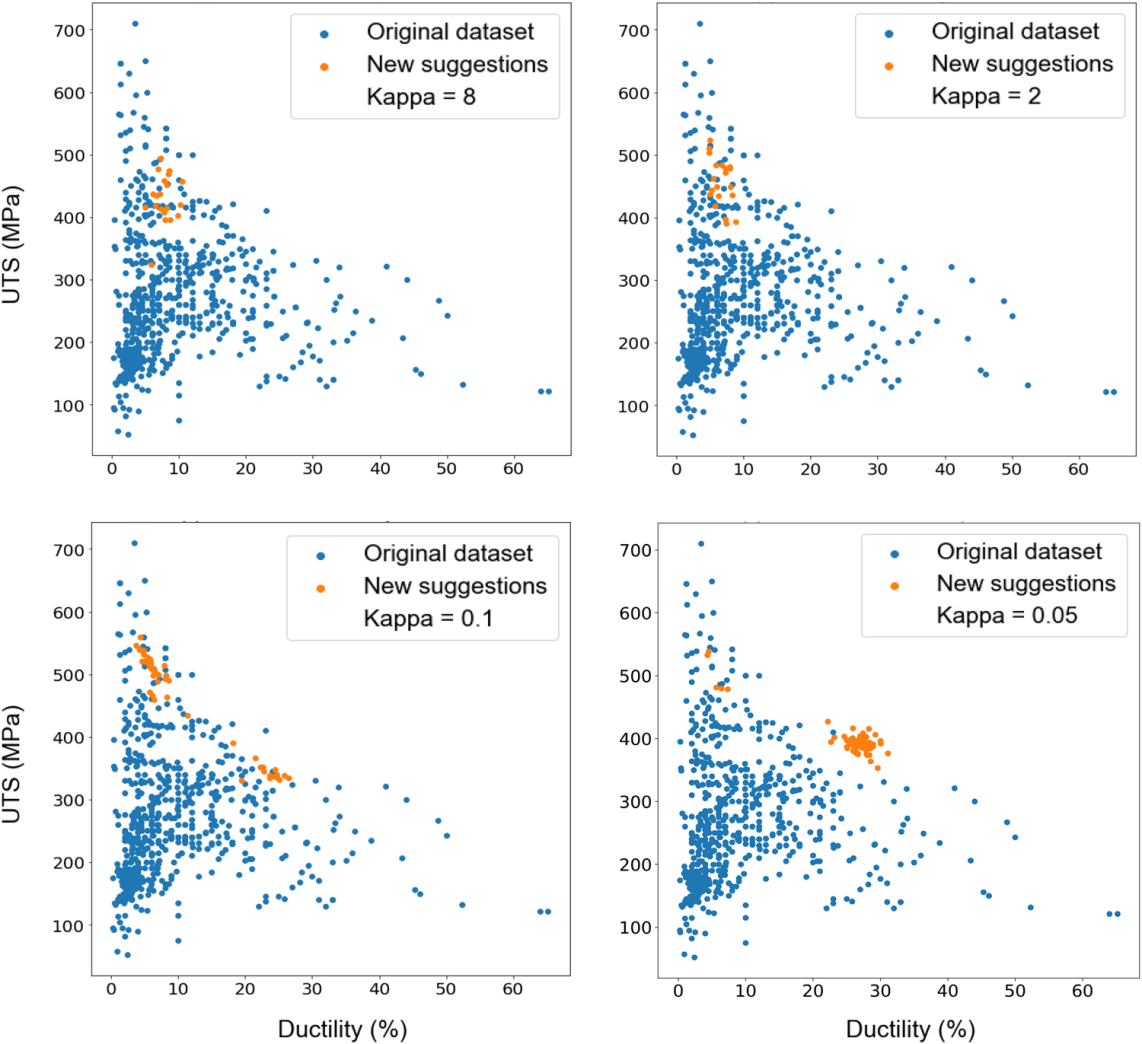
The effect of the hyperparameter kappa in the trade-off between exploration and exploitation in the process of proposing new Mg alloys by Bayesian optimisation. Orange dots are the new suggestions via the optimisation, and blue dots are the original dataset of existing alloys. Maximising the mechanical properties of UTS and ductility as a multi-objective optimisation problem is defined in the UCB acquisition function.

Within the search for the global optimum, the optimiser balances the exploration of promising regions and the exploitation of known optimal regions. Upper confidence bound is one of the popular strategies employed to manage this exploration–exploitation trade-off. As defined in Eq. (7), the kappa coefficient controls this trade-off that refers to the delicate balance between exploring new regions of the search space to discover potentially better solutions and exploiting the information gained from previous observations to focus on promising areas. In the context of Bayesian optimisation, this trade-off is crucial as it enables efficient exploration of the search space while converging to the optimal solution. The UCB algorithm has demonstrated remarkable performance in various optimisation tasks, including hyperparameter tuning, experimental design, and materials discovery. Its ability to effectively manage the exploration–exploitation trade-off makes it a popular choice for Bayesian optimisation applications. However, the appropriate tuning of the exploration parameter is crucial to strike the right balance, as overly aggressive exploration can lead to excessive sampling in unproductive regions, while overly conservative exploration can result in premature convergence to suboptimal solutions. The optimiser’s suggestions with various kappa values in the UCB method are shown in Fig. 7. New families of Mg alloys suggested by the optimiser are Mg–Ca–Gd–Ga, Mg–Al–Gd–Ga, Mg–Gd–Ga, Mg–Y–Gd–Ni–Ga, Mg–Gd–Li–Ni–Ga, and Mg–Gd–Yb–Ni–Ga (with their chemical composition (wt%) and production route provided in Table S1). It is noted that in this work, to keep the focus on technical facets, the cost of elements addition in each iteration is not included in optimisation (which can be highly variable and a challenge to quantify in a manner that is consistent over a period of years).

**Figure 8:**
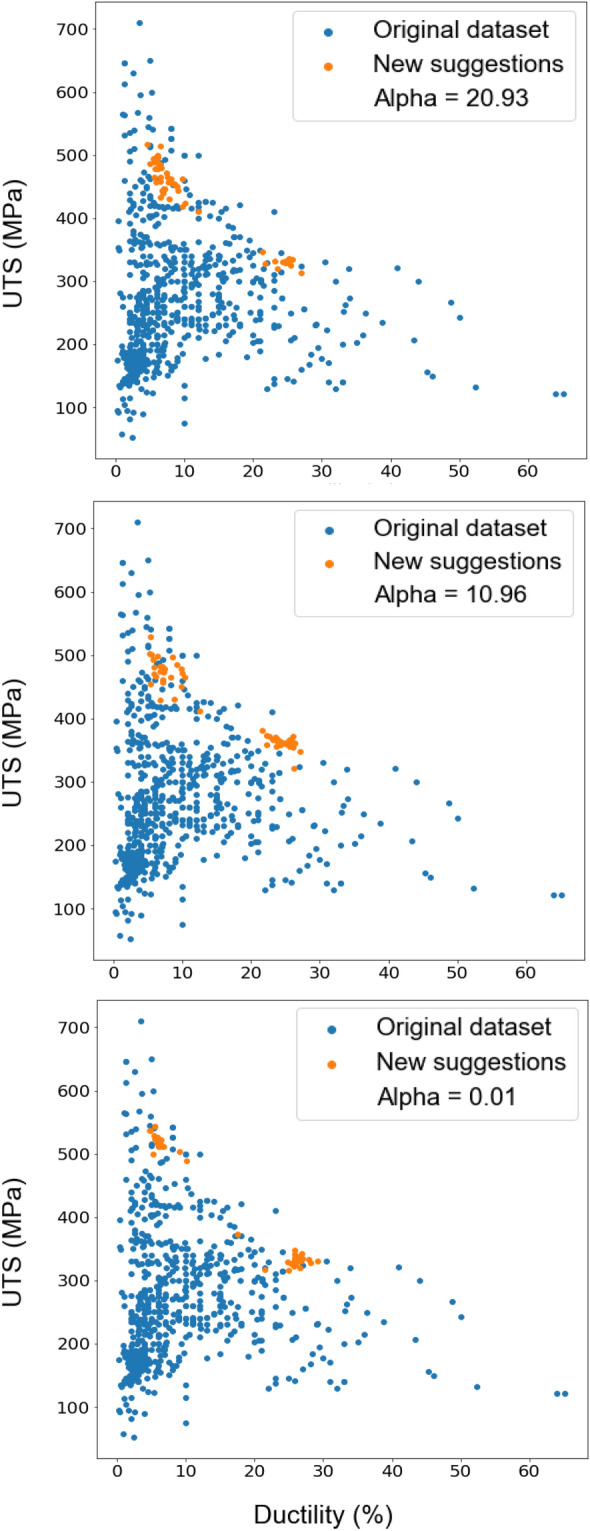
The effect of the hyperparameter alpha in the trade-off between exploration and exploitation in proposing new Mg alloys by Bayesian optimisation. Orange dots are the new suggestions by the optimisation and blue dots are the original dataset of existing alloys. Maximising mechanical properties of UTS and ductility as a multi-objective optimisation problem is defined in the MI acquisition function.

MI-based acquisition functions typically leverage the predictive distribution obtained from a surrogate model, here a Gaussian process, to estimate the MI. This distribution captures the uncertainty in the model's predictions, allowing the acquisition function to balance exploration and exploitation effectively. High uncertainty regions, where the model lacks confidence, were explored to gain information about potentially better solutions, while regions with low uncertainty, where the model is confident about high rewards, were exploited to refine the search around promising solutions. Compared to the UCB algorithm, MI-based acquisition functions offer several advantages. MI can capture more complex relationships between input variables and the objective function, making it particularly useful in high-dimensional and non-linear optimisation problems. Additionally, MI-based acquisition functions tend to exhibit smoother acquisition landscapes, leading to improved convergence and reduced sensitivity to the exploration parameter. The optimiser’s suggestions with various alpha values in the MI method are shown in Fig. 8. New families of Mg alloys suggested by the optimiser are Mg–Gd–Ni–Ga, Mg–Zn–Gd–Ga, Mg–Gd–Ga, Mg–Gd–Yb–Ga, and Mg–Gd–Li–Ga (with their chemical composition (wt%) and production route provided in Table S1).

### Multi-objective batch Bayesian optimisation

**Figure 9:**
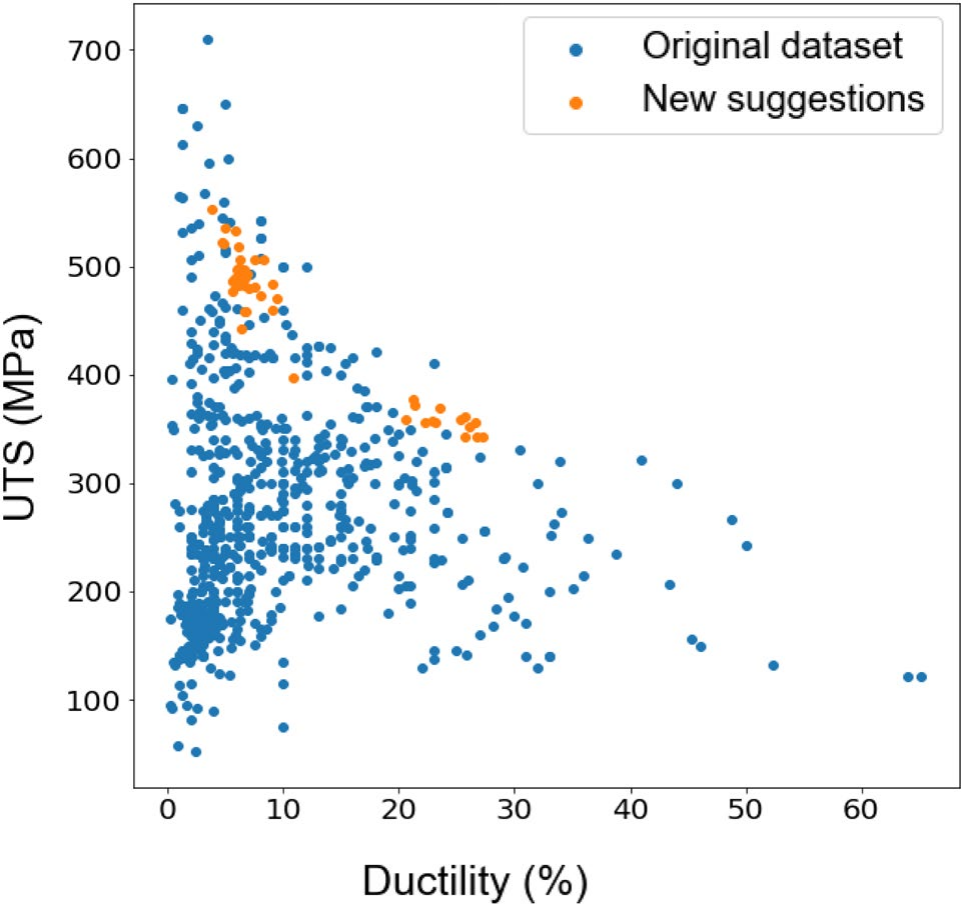
UTS versus ductility of the alloys suggested by batch Bayesian optimisation (orange dots) and original datapoints of existing alloys (blue dots). The batch size is set at 5 in a single run, and the process iterated 10 times.

The outcomes of the batch method implemented in a single, are presented in Fig. 9. The optimiser was trained using the existing Mg-alloy data, employing a batch size of 5 over 10 repetitions of searches. Proposed new compositions as a batch of alloys allow us to pick several samples in a single run. To validate the results and repeat the search, the batch of alloys can be fabricated and tested in parallel. Actual mechanical properties of samples can be added to the original training data. New families of Mg alloys suggested by the batch optimiser are Mg–Ca–Nd–Ga, Mg–Gd–Ga, Mg–Gd–Yb–Sb–Ga, Mg–Gd–Li–Ni–Ga, Mg–Gd–Li–Mn–Ga, Mg–Gd–Si–Ga, Mg–Y–Zn–Nd–Sr–Ga, and Mg–Gd–Er–Pr–Ga (with their chemical composition (wt%) and production route provided in Table S1).

## Data availability for digital alloy design

A graphical user interface (GUI) was designed that connects to the above-mentioned optimisers, with a user interactive tool and display for the proposed alloys. Users may interact with the alloy design tool via the web-based GUI and enter their desired range of compositions to be explored, along with the exploration of any potential thermomechanical processing. An image of the GUI menu is shown in Fig. S3. In the left column, the lower and upper bounds in weight percentage (wt%) of the chemical composition in terms of various elements can be defined. Preferred thermomechanical processes can be selected from the upper right section called “Heat Treatment”. Sampling size, number of suggested alloy “discoveries” to display, the maximum number of alloying elements in each alloy, the maximum sum of the alloying elements to be explored (in wt%), and the mechanical properties that the user is interested to optimise, make up the main parameters of the “Bayesian Optimisation Setting” section. After running the optimiser, the results (presented as a composition and accompanying thermomechanical treatment) will be shown.

The GitHub code associated with the present study, has been linked with this Google Collaboratory notebook to develop a user-interactive web tool that is publicly accessible through the following hyperlink: https://colab.research.google.com/drive/1wR0bQnxdAVUurH879dQNZ5wVzc0jGeD1#scrollTo=B5qe8hjnEyxe.

## Conclusions

The present study has provided the background, context, and tool development for digital alloy design and optimisation for Mg alloys. Specifically, optimisation is focused on the attainment of desirable mechanical properties including ultimate tensile strength and ductility. The Bayesian optimiser considers the data distribution via a probabilistic model that includes the function estimate and associated uncertainty around the estimation. New families of Mg alloys with maximised ultimate tensile strength (420–490 MPa) and ductility (12–30%) are capable of being generated *en masse*—based on a user interactive tool. In addition, the following conclusions may be drawn:

1. Active learning within the prescriptive analysis is less dependent on the quality and volume of training data, compared with predictive analysis.
2. The acquisition function can balance the exploration and exploitation, resulting in an efficient search process and optimal design.
3. Regret analysis provided an estimate of the performance of the optimiser and a means to validate optimised suggestions.
4. Bayesian optimisation was determined to be capable of optimising multiple properties simultaneously. The present study applies Bayesian optimisation to maximise either the Mg-alloy ductility, or ultimate tensile strength, or both the ductility and ultimate tensile strength simultaneously
5. The process of batch optimisation provided a number of optimised Mg-alloy suggestions (for target properties) in a single application of the model available as a public open access web tool.

### Supplementary Information

Supplementary Information.

## Data Availability

The codes developed in this study are openly available at the following site: https://colab.research.google.com/drive/1wR0bQnxdAVUurH879dQNZ5wVzc0jGeD1#scrollTo=B5qe8hjnEyxe. The datasets used and analysed during the current study available from the corresponding author on reasonable request.
