# Supplementary material for: An active machine learning approach for optimal design of magnesium alloys using Bayesian optimisation
Source: Sci Rep. 2024 Apr 9;14:8299. doi: 10.1038/s41598-024-59100-9 (PMC11004116; doi:10.1038/s41598-024-59100-9)
Supplement: Supplementary file 1 — Supplementary Information. [file 41598_2024_59100_MOESM1_ESM.docx]

**An active machine learning approach for optimal design of magnesium alloys using Bayesian optimisation**

M. Ghorbani^1,2,*^, M. Boley^3^, P.N.H. Nakashima^1^ and N. Birbilis^2,*^

^1^Department of Materials Science and Engineering, Monash University, VIC. 3800, Australia.

^2^Faculty of Engineering, Science and the Built Environment, Deakin University, VIC. 3125, Australia.

^3^Faculty of Information Technology, Monash University, VIC. 3800, Australia.

**Supplementary information:**

**Performance of the trained Gaussian regressor models**

A Gaussian regressor model as the surrogate function within the suggested Bayesian optimiser was trained with the whole Mg-alloy dataset. The parity plots (actual value in the X-axis and Gaussian probabilistic model predicted value in the Y-axis) along with the associated uncertainties are shown in Figures S1 and S2 for the target properties of strength and ductility, respectively.

**Figure S1**. *Predicted versus actual values of UTS for Mg alloys via Gaussian process regressor model (GPR) are shown in red dots. The associated uncertainties for the predicted test points are shown in green dots. The blue line indicates the ideal prediction values of UTS.*

**

**Figure S2**. *Predicted versus actual values of ductility for Mg alloys via Gaussian process regressor model (GPR) are shown in red dots. The associated uncertainties for the predicted test points are shown in green dots. The blue line indicates the ideal prediction values of ductility.*

**Mg-alloy design graphical user interface (GUI)**

A user-interactive web tool, an image of which is shown in Figure S3, is available for use in order to streamline the work associated with digital alloy design. The web tool serves to provide new candidate alloys – employing the workflow presented in this paper – for user-specified property targets and optimised Mg-alloy design. This digital design tool also affords an estimation of the mechanical properties for a number of optimal Mg-alloys within the pre-defined range of the composition and a variety of thermomechanical processing routes. Thermomechanical categories are limited to the selection of between one and six categories at a time. Furthermore, this tool also enables one to gain some intuition for the variation of mechanical properties with composition and the synergistic effects of certain alloying elements – as well as the effects of different thermomechanical processing routes. It is to be seen as a complement to experimental design and testing; however, the present approach may significantly streamline the alloy discovery and development process.

**Figure S3**. *The graphical user interface (GUI) of the Bayesian optimiser presented in this study. Users may define their preferred composition ranges (left columns) and the (one or more) desired thermomechanical processes (upper right section). The Bottom right section sets the optimiser’s parameters.*

**A selection of new families of Mg alloys suggested from Bayesian optimisation**

The chemical composition (wt.%) and production route of a selection of newly proposed alloys from the process of Bayesian optimisation are provided in Table S1.

**Table S1**. *The chemical composition and production route of some Mg-alloys proposed by Bayesian optimisation.*

| **Mg** | **Y** | **Al** | **Ca** | **Gd** | **Si** | **Tb** | **Li** | **Yb** | **Sr** | **Sb** | **Ni** | **Ga** | **Ag** | **Sc** | **Pr** | **Er** | **Bi** | **Production route** | **UTS(MPa)** | **Ductility (%)** |
| --- | --- | --- | --- | --- | --- | --- | --- | --- | --- | --- | --- | --- | --- | --- | --- | --- | --- | --- | --- | --- |
| 93.7 | 0.0 | 0.0 | 0.03 | 5.43 | 0.0 | 0.0 | 0.0 | 0.0 | 0.0 | 0.0 | 0.0 | 0.84 | 0.0 | 0.0 | 0.0 | 0.0 | 0.0 | Extruded | 324-405 | 26-40 |
| 93.72 | 0.0 | 0.0 | 0.43 | 4.82 | 0.3 | 0.0 | 0.0 | 0.0 | 0.0 | 0.0 | 0.0 | 0.73 | 0.0 | 0.0 | 0.0 | 0.0 | 0.0 | Extruded | 333-443 | 25-38 |
| 94.16 | 0.16 | 0.0 | 0.43 | 4.32 | 0.0 | 0.06 | 0.0 | 0.0 | 0.0 | 0.0 | 0.0 | 0.87 | 0.0 | 0.0 | 0.0 | 0.0 | 0.0 | Extruded | 320-434 | 27-39 |
| 93.31 | 0.0 | 0.0 | 0.4 | 5.67 | 0.0 | 0.0 | 0.0 | 0.0 | 0.0 | 0.0 | 0.0 | 0.62 | 0.0 | 0.0 | 0.0 | 0.0 | 0.0 | Extruded | 353-465 | 28-41 |
| 93.08 | 0.0 | 0.0 | 1.78 | 3.72 | 0.0 | 0.0 | 0.0 | 0.04 | 0.0 | 0.26 | 0.0 | 0.98 | 0.0 | 0.0 | 0.0 | 0.0 | 0.14 | Extruded | 385-551 | 23-31 |
| 94.14 | 0.0 | 0.0 | 0.30 | 4.3 | 0.0 | 0.0 | 0.0 | 0.0 | 0.0 | 0.0 | 0.3 | 0.96 | 0.0 | 0.0 | 0.0 | 0.0 | 0.0 | Extruded | 376-540 | 26-32 |
| 94.28 | 0.0 | 0.0 | 0.02 | 4.55 | 0.0 | 0.0 | 0.0 | 0.0 | 0.11 | 0.0 | 0.0 | 0.96 | 0.0 | 0.08 | 0.0 | 0.0 | 0.0 | Extruded | 375-540 | 26-30 |
| 93.69 | 0.0 | 0.0 | 1.48 | 4.02 | 0.0 | 0.0 | 0.0 | 0.0 | 0.0 | 0.0 | 0.0 | 0.81 | 0.0 | 0.0 | 0.0 | 0.0 | 0.0 | Extruded | 375-540 | 24-31 |
| 92.69 | 0.0 | 0.0 | 0.7 | 5.21 | 0.0 | 0.25 | 0.0 | 0.0 | 0.0 | 0.0 | 0.27 | 0.73 | 0.15 | 0.0 | 0.0 | 0.0 | 0.0 | Extruded | 371-538 | 23-32 |
| 94.16 | 0.0 | 0.0 | 0.24 | 4.19 | 0.0 | 0.0 | 0.0 | 0.64 | 0.0 | 0.0 | 0.0 | 0.77 | 0.0 | 0.0 | 0.0 | 0.0 | 0.0 | Extruded | 374-540 | 26-30 |
| 93.68 | 0.0 | 0.47 | 0.0 | 4.59 | 0.0 | 0.0 | 0.0 | 0.0 | 0.0 | 0.0 | 0.0 | 0.82 | 0.44 | 0.0 | 0.0 | 0.0 | 0.0 | Extruded | 382-548 | 26-30 |
| 94.36 | 0.0 | 0.06 | 0.0 | 4.29 | 0.0 | 0.0 | 0.0 | 0.0 | 0.0 | 0.0 | 0.0 | 0.78 | 0.0 | 0.0 | 0.51 | 0.0 | 0.0 | Extruded | 374-450 | 26-31 |
| 94.9 | 0.0 | 0.21 | 0.0 | 3.99 | 0.0 | 0.0 | 0.0 | 0.0 | 0.0 | 0.0 | 0.0 | 0.9 | 0.0 | 0.0 | 0.0 | 0.0 | 0.0 | Extruded | 392-568 | 24-36 |
| 94.77 | 0.0 | 0.09 | 0.0 | 3.76 | 0.0 | 0.0 | 0.0 | 0.0 | 0.0 | 0.0 | 0.88 | 0.46 | 0.0 | 0.04 | 0.0 | 0.0 | 0.0 | Extruded | 382-561 | 25-32 |
| 93.93 | 0.0 | 0.0 | 0.0 | 5.03 | 0.0 | 0.0 | 0.0 | 0.0 | 0.0 | 0.0 | 0.13 | 0.91 | 0.0 | 0.0 | 0.0 | 0.0 | 0.0 | Extruded | 336-454 | 24-39 |
| 94.54 | 0.0 | 0.0 | 0.0 | 4.61 | 0.0 | 0.0 | 0.0 | 0.0 | 0.0 | 0.0 | 0.0 | 0.85 | 0.0 | 0.0 | 0.0 | 0.0 | 0.0 | Extruded | 331-424 | 25-40 |
| 93.84 | 1.36 | 0.0 | 0.0 | 3.77 | 0.0 | 0.0 | 0.0 | 0.0 | 0.0 | 0.0 | 0.89 | 0.14 | 0.0 | 0.0 | 0.0 | 0.0 | 0.0 | Extruded | 321-420 | 27-38 |
| 93.25 | 1.87 | 0.0 | 0.0 | 4.16 | 0.0 | 0.07 | 0.0 | 0.0 | 0.0 | 0.0 | 0.65 | 0.0 | 0.0 | 0.0 | 0.0 | 0.0 | 0.0 | Extruded | 316-421 | 26-37 |
| 93.67 | 0.0 | 0.0 | 0.0 | 4.33 | 0.0 | 0.0 | 0.48 | 0.0 | 0.0 | 0.0 | 0.61 | 0.91 | 0.0 | 0.0 | 0.0 | 0.0 | 0.0 | Extruded | 372-540 | 26-31 |
| 92.93 | 0.0 | 0.0 | 0.0 | 5.31 | 0.0 | 0.0 | 0.0 | 0.12 | 0.0 | 0.0 | 0.68 | 0.96 | 0.0 | 0.0 | 0.0 | 0.0 | 0.0 | Extruded | 358-455 | 25-38 |
| 93.09 | 0.0 | 0.0 | 0.0 | 4.50 | 0.0 | 0.0 | 0.0 | 0.06 | 0.0 | 0.0 | 0.95 | 0.85 | 0.33 | 0.0 | 0.0 | 0.22 | 0.0 | Extruded | 335-455 | 26-37 |
| 93.66 | 0.0 | 0.0 | 0.0 | 5.30 | 0.0 | 0.0 | 0.0 | 0.07 | 0.0 | 0.0 | 0.05 | 0.88 | 0.0 | 0.0 | 0.0 | 0.0 | 0.04 | Extruded | 326-411 | 26-40 |
| 94.31 | 0.0 | 0.0 | 0.0 | 4.41 | 0.0 | 0.0 | 0.0 | 0.27 | 0.0 | 0.0 | 0.11 | 0.85 | 0.05 | 0.0 | 0.0 | 0.0 | 0.0 | Extruded | 376-544 | 26-40 |
